# Transcriptional Induction of Metallothionein by Tris(pentafluorophenyl)stibane in Cultured Bovine Aortic Endothelial Cells

**DOI:** 10.3390/ijms17091381

**Published:** 2016-08-23

**Authors:** Tomoya Fujie, Masaki Murakami, Eiko Yoshida, Shuji Yasuike, Tomoki Kimura, Yasuyuki Fujiwara, Chika Yamamoto, Toshiyuki Kaji

**Affiliations:** 1 Department of Environmental Health, Faculty of Pharmaceutical Sciences, Tokyo University of Science, 2641 Yamazaki, Noda 278-8510, Japan; t-fujie@phar.toho-u.ac.jp (T.F.); j3b13674@ed.tus.ac.jp (M.M.); eyoshida@rs.tus.ac.jp (E.Y.); 2 Laboratory of Organic and Medicinal Chemistry, School of Pharmaceutical Sciences, Aichi Gakuin University, 1-100 Kusumoto-cho, Chikusa-ku, Nagoya 464-8650, Japan; 3 Depertment of Life Science, Faculty of Science and Engineering, Setsunan University, 17-8 Ikedanakamachi, Neyagawa 572-8508, Japan; tomoki@lif.setsunan.ac.jp; 4 Department of Environmental Health, School of Pharmacy, Tokyo University of Pharmacy and Life Sciences, 1432-1 Horinouchi, Hachioji 192-0392, Japan; yasuyuki@toyaku.ac.jp; 5 Department of Environmental Health, Faculty of Pharmaceutical Sciences, Toho University, 2-2-1 Miyama, Funabashi 274-8510, Japan; yamamoto@phar.toho-u.ac.jp

**Keywords:** bio-organometallics, metallothionein, vascular endothelial cell, organic–inorganic hybrid molecule, organoantimony compound

## Abstract

Vascular endothelial cells cover the luminal surface of blood vessels and contribute to the prevention of vascular disorders such as atherosclerosis. Metallothionein (MT) is a low molecular weight, cysteine-rich, metal-binding, inducible protein, which protects cells from the toxicity of heavy metals and active oxygen species. Endothelial MT is not induced by inorganic zinc. Adequate tools are required to investigate the mechanisms underlying endothelial MT induction. In the present study, we found that an organoantimony compound, tris(pentafluorophenyl)stibane, induces gene expression of *MT-1A* and *MT-2A*, which are subisoforms of MT in bovine aortic endothelial cells. The data reveal that *MT-1A* is induced by activation of both the MTF-1–MRE and Nrf2–ARE pathways, whereas *MT-2A* expression requires only activation of the MTF-1–MRE pathway. The present data suggest that the original role of MT-1 is to protect cells from heavy metal toxicity and oxidative stress in the biological defense system, while that of MT-2 is to regulate intracellular zinc metabolism.

## 1. Introduction

Metallothionein (MT) is a low molecular weight, cysteine-rich, metal-binding, and inducible protein, which was found as a protein containing cadmium and zinc from equine renal cortex [1,2]. There are four isoforms of MT—MT-1, MT-2, MT-3, and MT-4—in mammals [3], and MT-1 consists of several subisoforms: seven in human tissue but only two—MT-1A and MT-1E— in bovine tissue. Among MT isoforms, MT-3 and MT-4 exist in specific tissue: MT-3 is in the neural tissue [4] and MT-4 is in stratified squamous epithelia [3]. MT-1 and MT-2 ubiquitously exist in the liver, kidney, and other organs and are induced by heavy metals such as cadmium and zinc, oxidative stress, and other physiological factors including cytokines and growth factors [2,5,6]. MT-1 and MT-2 are considered to have the same functions and induction mechanisms [7,8]. However, several reports show the induction level is different between MT-1 and MT-2 [9,10,11,12,13,14], suggesting that these MT isoforms may be induced differently.

Mechanisms underlying transcriptional induction of *MT* genes are not completely understood. Metal response element-binding transcription factor-1 (MTF-1) mediates heavy metal signaling and is essentially required for *MT* gene expression [15]. MTF-1 is activated by zinc and binds the sequences termed metal responsive element (MRE) that exist in the upstream region of the *MT* gene [16,17]. Additionally, the *MT* gene includes sequences termed antioxidant response element (ARE) in the promoter region [2,18], which are activated by the transcriptional factor nuclear factor-erythroid 2-related factor 2 (Nrf2) and regulate the expression of antioxidant genes [19]. Although ARE is involved in the transcriptional induction of *MT* genes by hydrogen peroxide [20], there is little information about the role of ARE in the MT induction. We hypothesize that the MTF-1–MRE and Nrf2–ARE pathways cooperatively regulate transcription of MT.

Vascular endothelial cells cover the luminal surface of blood vessels and prevent vascular disorders such as atherosclerosis by regulating the blood coagulation-fibrinolytic system and vascular tonus [21]. In addition, heavy metals may affect the vascular endothelial cell functions, which can modify the organ toxicity [22]. In fact, it has been reported that heavy metals such as cadmium and mercury cause a progression of vascular disorders [23,24]. Since MT protects cells from heavy metal toxicity [25] and oxidative stress [26], MT is considered a multifunctional protein involved in defense mechanisms. We previously studied the toxicity of heavy metals in cell culture vascular endothelial cells and found that cadmium induces MT in vascular endothelial cells and in other cell types whereas zinc—a representative MT inducer—does not induce MT in vascular endothelial cells in a serum-free medium [27,28], although the metal at high concentrations can induce MT in medium containing serum [29,30]. In addition, endothelial MT is not induced only by activation of the MTF-1–MRE pathway [28]. Therefore, inorganic zinc is not a good tool to clarify the mechanisms underlying endothelial MT induction. We hypothesized that organometallic compounds could act as good tools for analyzing endothelial MT induction as described below.

Organic-inorganic hybrid molecules are composed of an organic structure and metal(s) and are in general used as reagents in chemical synthetic reactions, since pioneers such as Grignard and Wittig used the molecules as organic synthesis reagents [31,32]. Studies on organopnictogen compounds, a type of organic-inorganic hybrid molecules, indicate that their cytotoxicity depends on intracellular accumulation and is influenced by intramolecular metal(s), organic structure, and the interaction between the metal(s) and the structure [33,34]. Organic-inorganic hybrid molecules may be useful for analyzing the mechanisms underlying endothelial MT induction. We refer to the strategy of using organic-inorganic hybrid molecules as tools to analyze biological systems as “bio-organometallics”. In the present study, we constructed a library of 28 organoantimony compounds and tested the effect on transcriptional induction of *MT* genes and found that tris(pentafluorophenyl)stibane (termed Sb35) induces the gene expression of the subisoforms *MT-1A* and *MT-2A* in bovine aortic endothelial cells. We analyzed the intracellular pathways involved in endothelial MT induction using Sb35.

## 2. Results

### 2.1. Transcriptional Induction of MT Isoforms by Sb35

**Figure 1:**
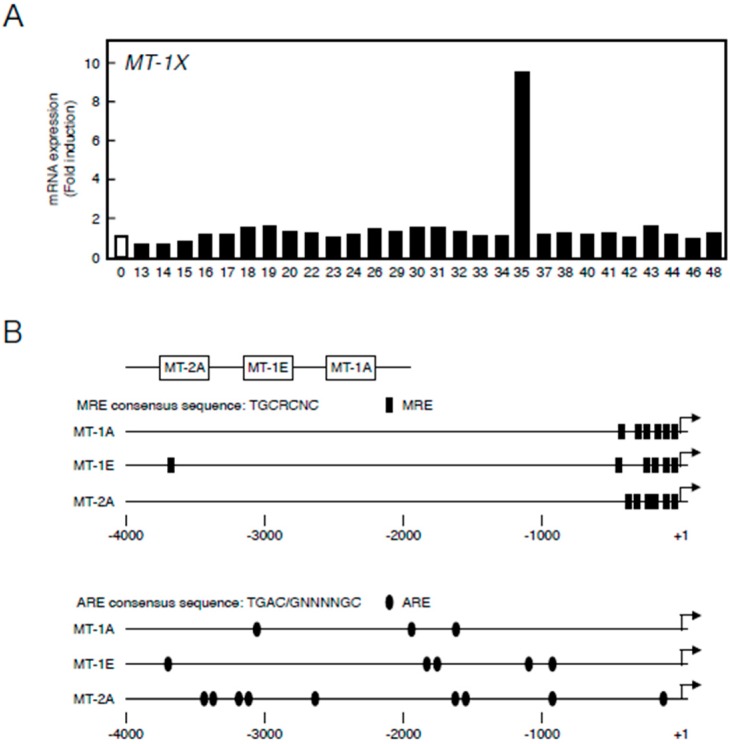
(**A**) Transcriptional induction of *MT-1X* in vascular endothelial cells after treatment with organoantimony compounds shown in Table 1. Human brain microvascular endothelial cells were incubated with the organoantimony compounds at 10 µM each for 3 h, and the expression of *MT-1X* mRNA was determined by real-time RT-PCR; (**B**) The map of MRE and ARE regions in the bovine MT promoter.

First, we constructed a library of 28 organoantimony compounds (Table 1). We used human vascular endothelial cells in the first experiment to confirm that *MT* induction by organoantimony compound(s) is possible in human endothelial cells as well as in bovine endothelial cells as described below. We tested the induction of expression of *MT-1X*—the major MT isoforms in human vascular endothelial cells [30]. As shown in Figure 1A, it was found that Sb35 induces high *MT-1X* gene expression. As stated below, As35 and P35 as well as Sb35 increased the expression of *MT* mRNAs, suggesting that the molecular structure of these hybrid molecules is required for the transcriptional induction of endothelial MT. We predict that antimony compounds, which have no ability to induce *MT* mRNA expression, lack the required molecular structure.

**Figure 2:**
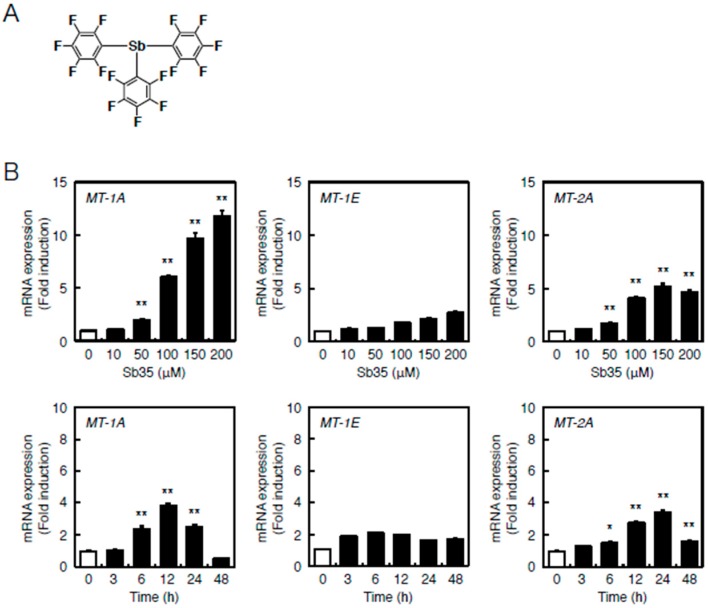
Sb35-induced transcription of endothelial *MT-1A*, *MT-1E*, and *MT-2A*. (**A**) Structure of Sb35; (**B**) Sb35-induced transcription of MT. Vascular endothelial cells were incubated with or without 10, 50, 100, 150, or 200 µM Sb35 for 12 h (**upper** panels) or 100 µM Sb35 for 3, 6, 12, 24, or 48 h (**lower** panels). *MT-1A*, *MT-1E*, and *MT-2A* mRNA expression was determined by performing real-time RT-PCR. Data are expressed as the mean ± SE of three representative samples, with each sample obtained from three independent experiments. * *p* < 0.05 and ** *p* < 0.01 indicate significantly different from the corresponding control.

Figure 1B depicts the locations of MREs and AREs in the upstream regions of *MT* genes of the bovine cells. Bovine cells express three MT subisoforms—MT-1A, MT-1E, and MT-2A—and each of their genes has MRE and ARE consensus sequences in the promoter region. The number of MT-1 subisoforms is eight in human and two in bovine cells. To investigate the difference in the intracellular signaling between MT-1 and MT-2 isoforms, the subsequent experiments were performed using bovine endothelial cells having only two subisoforms of MT-1. In bovine aortic endothelial cells, Sb35 (Figure 2a) induced *MT-1A* and *MT-2A* gene expression in a concentration-dependent manner when treated for 12 h (Figure 2b, upper panels). We observed maximum induction of *MT-1A* and *MT-2A* by 100 µM Sb35 at 12 and 24 h, respectively (Figure 2b, lower panels), indicating that Sb35 stimulates the transcriptional induction of *MT-1* and *MT-2* isoform genes in the cells; decrease in the induction at 48 h is possibly due to the metabolism of Sb35 and/or downregulation of the intracellular signaling activated by Sb35, although the details are unclear. However, induction of MT protein by Sb35 was not observed in Western blot analysis (Figure S1), suggesting that Sb35 compound is a tool to analyze the transcriptional induction of endothelial *MT* but not an agent for MT induction to protect cells from the toxicity of heavy metals and generated reactive oxygen species. In addition, Sb35 did not generate reactive oxygen species (Figure S2), suggesting that the transcriptional induction of *MT* by Sb35 is not mediated by reactive oxygen species. In addition, *MT-1A*, *MT-1E*, and *MT-2A* mRNA levels increased by approximately 12, three, and six fold, respectively, after Sb35 treatment. On the other hand, cadmium, a typical MT inducer, increased *MT-1A*, *MT-1E*, and *MT-2A* mRNA levels by approximately 1000-, 15-, and 15-fold, respectively, suggesting that Sb35 induced MT more weakly than cadmium. This appears to be the reason why Sb35 cannot induce MT at the protein level.

### 2.2. Involvement of the MTF-1–MRE Pathway

**Figure 3:**
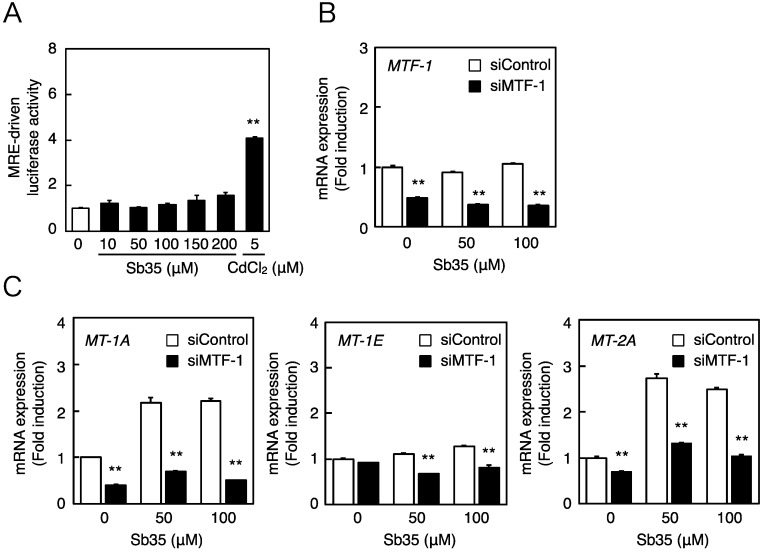
The MTF-1–MRE pathway mediates Sb35-induced transcription of endothelial *MT-1A*, *MT-1E*, and *MT-2A*. (**A**) MRE-driven transcriptional activity. Vascular endothelial cells transfected with an MRE reporter vector were treated with or without 10, 50, 100, 150, or 200 µM Sb35 or 5 µM cadmium for 12 h, and MRE-driven transcriptional activity was determined by performing the MRE-directed reporter assay; (**B**) siRNA-mediated *MTF-1* knockdown. Vascular endothelial cells transfected with control or *MTF-1* siRNA were treated with or without 50 or 100 µM Sb35 for 12 h, and expression of *MTF-1* mRNA was determined by performing real-time RT-PCR. Data are expressed as the mean ± SE of three samples. ** *p* < 0.01 indicates significantly different from the corresponding siControl; (**C**) Sb35-induced transcription of endothelial *MT-1A*, *MT-1E*, and *MT-2A* after *MTF-1* knockdown. Vascular endothelial cells transfected with control or *MTF-1* siRNA were treated with or without 50 or 100 µM Sb35 for 12 h. Data are expressed as the mean ± SE of three representative samples, with each sample obtained from three independent experiments. ** *p* < 0.01 indicates significantly different from the corresponding siControl.

Next, we investigated whether the MTF-1–MRE pathway was involved in Sb35-induced transcription of endothelial *MT* isoforms. Expression of MTF-1 was detected by real-time RT-PCR because we failed to detect bovine MTF-1 protein by Western blot analysis. Sb35 only slightly increased the MRE-driven transcriptional activity (Figure 3A). In the MTF-1 knockdown (Figure 3B), Sb35-induced expression of *MT-1A* and *MT-2A* was suppressed (Figure 3C), suggesting that the constitutive expression and activity of MTF-1 is sufficient for transcriptional induction of endothelial *MT-1A* and *MT-2A*. Sb35 did not induce the transcription of *MT-1E*, although siRNA-mediated knockdown of MTF-1 suppressed this constitutive expression. As reported previously [35], the MTF-1–MRE pathway is involved in Sb35 induction of endothelial *MT-1A* and *MT-2A*.

### 2.3. Involvement of the Nrf2–ARE Pathway

**Figure 4:**
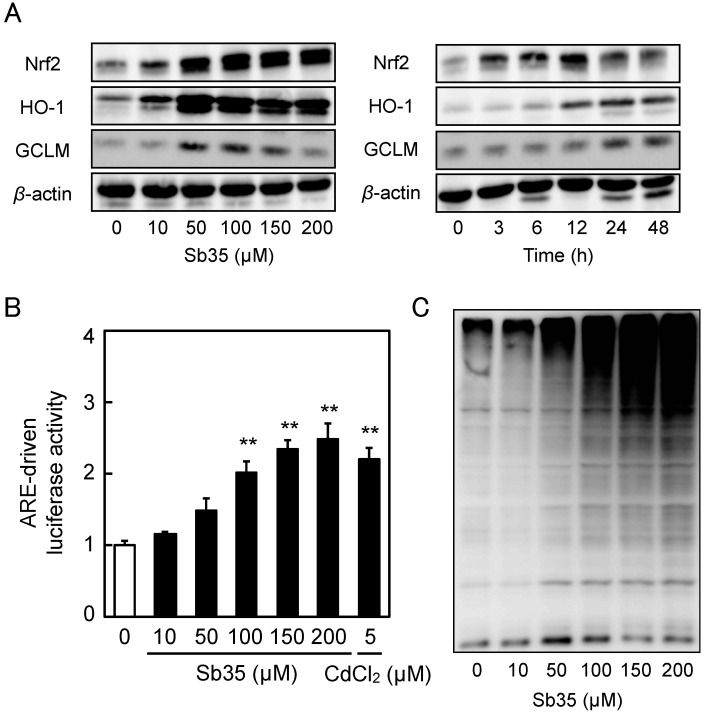
Expression of Nrf2 and its downstream proteins, Sb35-induced ARE-driven transcriptional activity, and Sb35-induced proteasome inhibition in vascular endothelial cells. (**A**) Expression of Nrf2, HO-1, and GCLM. Vascular endothelial cells were treated with or without 10, 50, 100, 150, or 200 µM Sb35 for 12 h (**left** panels) or 100 µM Sb35 for 3, 6, 12, 24, or 48 h (**right** panels), and expression of Nrf2, HO-1, and GCLM was determined by performing Western blotting; (**B**) ARE-driven transcriptional activity. Vascular endothelial cells transfected with an ARE reporter vector were treated with or without 10, 50, 100, 150, or 200 µM Sb35 or 5 µM cadmium for 12 h, and ARE-driven transcriptional activity was determined by performing ARE-directed reporter assay. Data are represented as mean ± SE of three representative samples, with each sample obtained from three independent experiments. ** *p* < 0.01 indicates significantly different from the corresponding control; (**C**) Sb35-induced proteasome inhibition. Vascular endothelial cells were treated with or without 10, 50, 100, 150, or 200 µM Sb35 for 24 h, and ubiquitinated proteins were determined by performing Western blotting.

Sb35 increased the intracellular accumulation of Nrf2 in a concentration- and time-dependent manner and upregulated Nrf2 target proteins such as HO-1 and GCLM (Figure 4A). Sb35 significantly increased the ARE-driven transcriptional activity in a concentration-dependent manner (Figure 4B), confirming that it activates the Nrf2–ARE pathway in vascular endothelial cells. It was shown that Sb35 exhibited proteasome inhibitory activity in a concentration-dependent manner (Figure 4C), suggesting that activation of Nrf2 by Sb35 is at least partly due to the proteasome inhibition that stabilizes Nrf2. Since Sb35 did not generate reactive oxygen species (Figure S2), it is unlikely that activation of Nrf2 by Sb35 is mediated by reactive oxygen species.

**Figure 5:**
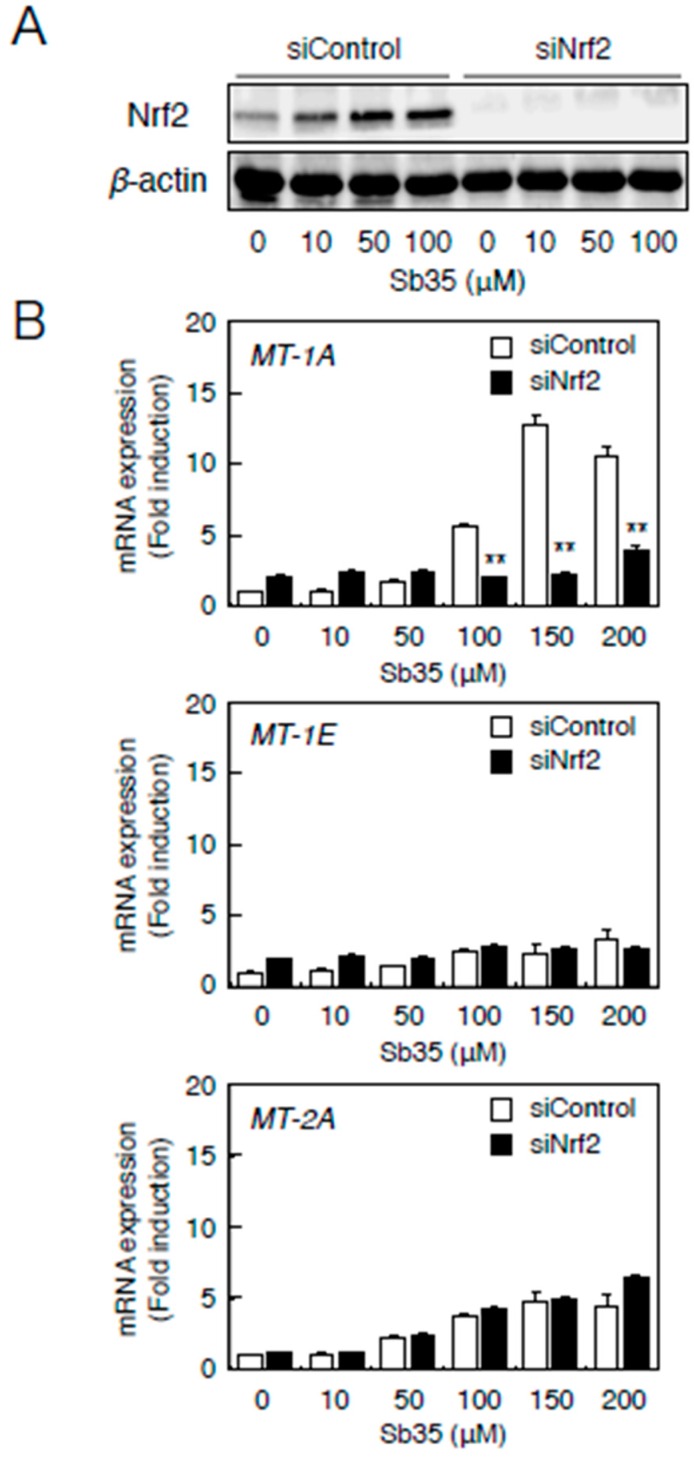
The Nrf2–ARE pathway mediates Sb35-induced transcription of endothelial *MT-1A*, *MT-1E*, and *MT-2A*. (**A**) Expression of Nrf2. Vascular endothelial cells transfected with control or Nrf2 siRNA were treated with or without 10, 50, or 100 µM Sb35 for 24 h, and expression of Nrf2 was determined by performing Western blotting; (**B**) Sb35-induced transcription of endothelial *MT-1A*, *MT-1E*, and *MT-2A* after *Nrf2* knockdown. Vascular endothelial cells transfected with control or *Nrf2* siRNA were treated with or without 10, 50, 100, 150, or 200 µM Sb35 for 12 h. Data are expressed as the mean ± SE of three representative samples, with each sample obtained from three independent experiments. ** *p* < 0.01 indicates significantly different from the corresponding siControl.

Nrf2 knockdown (Figure 5A) resulted in a markedly lower expression of *MT-1A* in the presence of Sb35 (Figure 5b, upper panel). In contrast, *MT-2A* expression in the Nrf2 knockdown and in the presence of Sb35 was unaffected (Figure 5B, lower panel). Sb35 did not induce the transcription of *MT-1E* (Figure 5b, middle panel). Thus, the transcriptional induction of *MT-1A* is regulated by both the MTF-1–MRE and Nrf2–ARE pathways whereas that of *MT-2A* is stimulated by only the MTF-1–MRE pathway in vascular endothelial cells.

### 2.4. Determination of the Pathway Involved in the Transcriptional Induction of Endothelial MT Using Tris(pentafluorophenyl)phosphane (P35)

**Figure 6:**
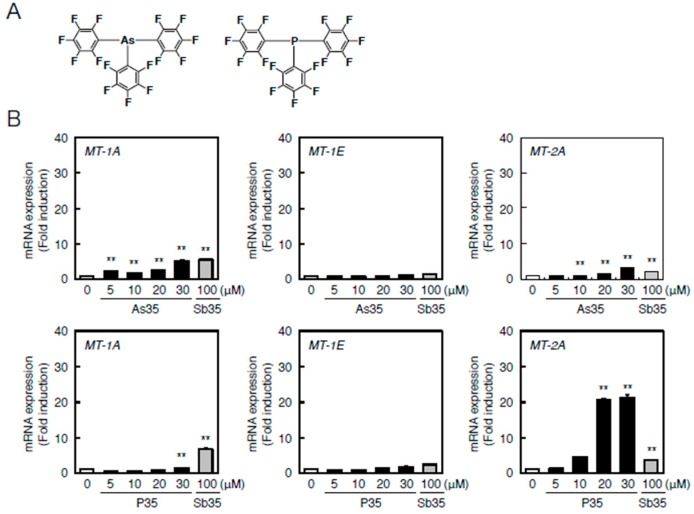
As35 or P35-induced transcription of endothelial *MT-1A*, *MT-1E*, and *MT-2A*. (**A**) Structures of As35 and P35; (**B**) Endothelial *MT-1A*, *MT-1E*, and *MT-2A* expression after As35 (**upper** panels) or P35 (lower panels) treatment. Vascular endothelial cells were treated with or without 10, 20, 50, or 100 µM As35 for 12 h (upper panels) or 5, 10, 20, or 30 µM P35 or 12 h (**lower** panels). The cells were also treated with 100 µM Sb35, which served as a comparative control. *MT-1A*, *MT-1E*, and *MT-2A* mRNA expression was determined by performing real-time RT-PCR. Data are expressed as the mean ± SE of three representative samples, with each sample obtained from three independent experiments. ** *p* < 0.01 indicate significantly different from the corresponding control.

Understanding the role of the antimony atom in Sb35 molecules in endothelial MT induction is important for understanding the bioactivity of Sb35 as a hybrid molecule. In order to increase the understanding of the role of antimony atom, we next investigated the effects of pnictogen analogues—As35 and P35 (Figure 6A)—on the transcriptional induction of *MT-1A*, *MT-1E*, and *MT-2A* in bovine aortic endothelial cells. As35 significantly increased *MT-1A* and *MT-2A* expression similarly to Sb35 (Figure 6B, upper panels). The transcriptional induction of *MT-1A* and *MT-1E* by P35 was very weak; however, P35 strongly induced transcription of *MT-2A* (Figure 6B, lower panels).

**Figure 7:**
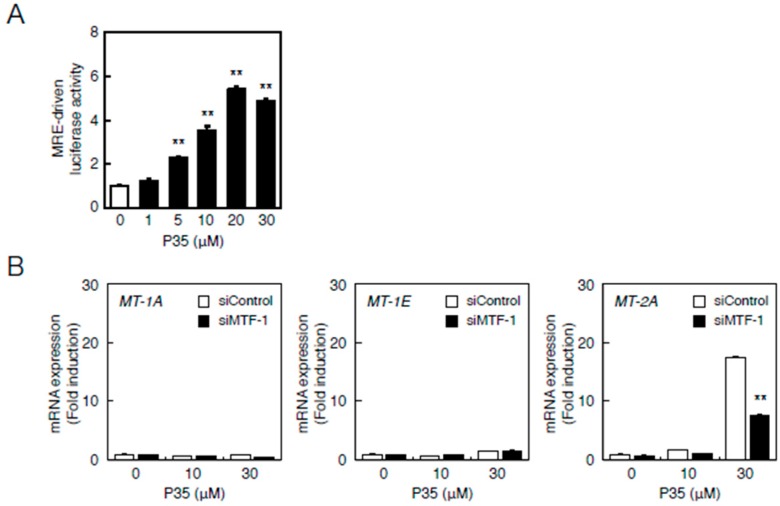
The MTF-1–MRE pathway mediates P35-induced transcription of endothelial *MT-1A*, *MT-1E*, and *MT-2A*. (**A**) Vascular endothelial cells transfected with an MRE reporter vector were treated with or without 1, 5, 10, 20, or 30 µM P35 for 12 h, and MRE-driven transcriptional activity was determined by performing the MRE-directed reporter assay. ** *p* < 0.01 indicates significantly different from the corresponding control; (**B**) P35-induced transcription of endothelial *MT-1A*, *MT-1E*, and *MT-2A* after *MTF-1* knockdown. Vascular endothelial cells transfected with control or *MTF-1* siRNA were treated with or without 10 or 30 µM P35 for 12 h. Data are expressed as the mean ± SE of three representative samples, with each sample obtained from three independent experiments. ** *p* < 0.01 indicates significantly different from the corresponding siControl.

Therefore, we investigated the involvement of the MTF-1–MRE and Nrf2–ARE pathways in the transcriptional induction of *MT-2A* by P35. P35 significantly increased MRE-driven transcription in a concentration-dependent manner in vascular endothelial cells (Figure 7A). The transcriptional induction of *MT-2A* by P35 was suppressed in a siRNA-mediated knockdown of MTF-1 (Figure 7B).

**Figure 8:**
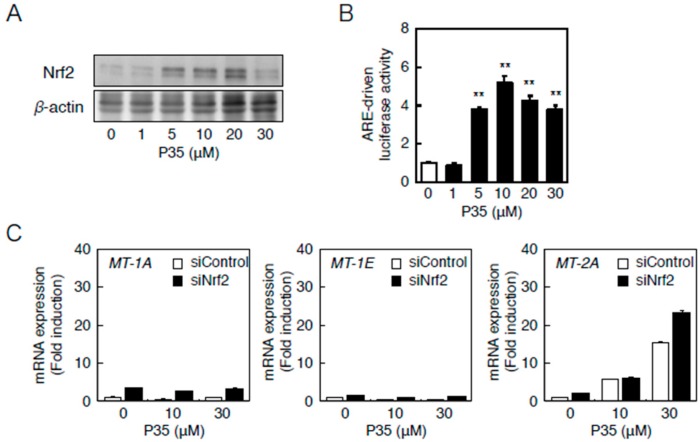
The Nrf2–ARE pathway mediates P35-induced transcription of endothelial *MT-2A*. (**A**,**B**) ARE-driven transcriptional activity. Vascular endothelial cells transfected with an ARE reporter vector were treated with or without 1, 5, 10, 20, or 30 µM P35 for 12 h, and ARE-driven transcriptional activity was determined by performing the ARE-directed reporter assay. Data are expressed as the mean ± SE of three representative samples, with each sample obtained from three independent experiments. ** *p* < 0.01 indicates significantly different from the corresponding control; (**C**) P35-induced transcription of endothelial *MT-1A*, *MT-1E*, and *MT-2A* after *Nrf2* knockdown. Vascular endothelial cells transfected with control or *Nrf2* siRNA were treated with or without 10 or 30 µM P35 for 12 h. Data are expressed as the mean ± SE of three representative samples, with each sample obtained from three independent experiments. ** *p* < 0.01 indicates significantly different from the corresponding siControl.

Similarly, P35 activated Nrf2 (Figure 8A) and significantly increased ARE-driven transcription in a concentration-dependent manner (Figure 8B). The transcriptional induction of *MT-2A* by P35 was, however, unaffected in a siRNA-mediated knockdown of Nrf2 (Figure 8B), indicating that the Nrf2–ARE pathway does not transactivate *MT-2A* induction.

## 3. Discussion

MT protects cells from heavy metal toxicity and oxidative stress; however, the mechanisms underlying MT induction are not fully understood. Vascular endothelial cells regulate the blood coagulation-fibrinolytic system and vascular tone while MT likely protects the cells from functional damage, thereby preventing vascular disorders. In fact, fetal high-density lipoprotein, which is associated with the inhibition of atherosclerosis, reduces the expression of *MT-1X* and *MT-2A* in human placental endothelial cells [36]. Since zinc is not an inducer of endothelial MT synthesis [26,27], we used a library of organoantimony compounds to analyze the mechanisms of endothelial *MT* induction. We found that the organoantimony compound Sb35 causes transcriptional induction of *MT* isoforms—*MT-1A* and *MT-2A*—in bovine aortic endothelial cells. Among these MT isoforms, the transcriptional induction of *MT-1A* is regulated by either the MTF-1–MRE or Nrf2–ARE pathways whereas only the MTF-1–MRE pathway regulates *MT-2A* expression. Since sulforaphane, an Nrf2 activator, weakly induces MT isoform expression (data not shown), the MTF-1–MRE pathway is likely essential for transcriptional induction of all endothelial MT isoforms while the Nrf2–ARE pathway enhances the induction of *MT-1A* isoform by MTF-1. However, the increase in MRE-driven promoter activity by Sb35 was not so marked. Although the details are not clear, an assumption can be made that Sb35 may activate other regulatory pathways that induce transcription of *MT* genes [36] in vascular endothelial cells. Since Nrf2 is a transcriptional factor that induces the expression of antioxidant proteins such as HO-1 [37] and GCLM [38], the original role of MT-1A may be cytoprotection against oxidative damage by injury or inflammation. Alternatively, that of MT-2A may regulate zinc metabolism as well as mediate cytoprotection because the transcriptional induction requires only activation of MTF-1 by zinc. Specifically, MT-1 and MT-2 do not share these roles but the original roles may be different. A recent study suggested that MT-1, compared to MT-2, has a greater ability to bind cadmium, whereas MT-2 has higher ability to bind to zinc compared to MT-1 [39]. In addition, it was reported that cadmium increased the expression of only three genes, including *MT-1E*, *MT-1H*, and *MT-1B*, in human coronary artery endothelial cells by microarray analysis [35]. These reports partly support our hypothesis. Involvement of the Nrf2–ARE pathway in the transcriptional induction of *MT-1A* but not *MT-2A* is a new finding of the present study.

Recently, we found that the Nrf2–ARE pathway is involved in the cadmium mediated induction of endothelial MT isoforms including *MT-1E* and *MT-2A* [40]. Cadmium modifies Kelch-like ECH-associated protein 1, a negative regulator of Nrf2, and activates Nrf2, which is recruited to ARE1 and ARE5 of the promoter region of endothelial *MT-2A*. However, induction of both *MT-2A* and *MT-1E* is reinforced by ARE activation. Since *MT-1E* is located next to *MT-2A* in the genome, we speculated that recruitment of Nrf2 to the AREs of the *MT-2* promoter region stimulates the transcriptional activity of *MT-1* and *MT-2A*. In the present study, the Nrf2–ARE pathway increases the induction of *MT-1A* and *MT-1E* but not *MT-2A*. Although we have not identified AREs to which Nrf2 is recruited in the presence of Sb35, we speculate that Sb35 modifies Nrf2 and alters AREs to be recruited. For example, Nrf2 might be recruited to the promoter region of *MT-1* rather than that of *MT-2* in the presence of Sb35. In fact, Nrf2 is regulated by acetylation [41]. Proteasome inhibition results in Nrf2 stabilization [42] and acetylation [43]. We confirmed that in bovine aortic endothelial cells after 12 h Sb35 (>50 µM) inhibits proteasome activity whereas cadmium (<10 µM) does not [44]. It is likely that the degree of acetylation of Nrf2 is responsible for the differences in involvement of the Nrf2–ARE pathway in the *MT* isoform transcriptional induction between Sb35 and cadmium, although other mechanisms might also be involved.

P35’s induction of endothelial *MT-2A* transcription is unaffected by the Nrf2–ARE pathway. If P35-activated Nrf2 is recruited to AREs in the promoter regions of *MT-1A/E* genes, transcription of *MT-1A/E* would be stimulated, as is the case when Sb35 is present. Even if Nrf2 is recruited to *MT-2A* AREs, transcription of *MT-1A/E* would be stimulated, as is the case for cadmium. Although it is not known why P35-activated Nrf2 does not stimulate *MT-1A/E* transcription, the present data suggest that there is a mechanism by which only MT-2A is induced in vascular endothelial cells. In other words, both Sb35 and P35 activate the Nrf2–ARE pathway but only Sb35 may be able to act on the mechanism that is required for *MT-1A* gene expression. On the other hand, As35 increased the transcriptional induction of *MT-1A* and *MT-2A* similarly to Sb35, but at concentrations lower than that of Sb35. The properties of arsenic as an element are very similar to those of antimony. In our previous study, however, it was shown that intracellular accumulation of As35 was higher than that of Sb35 in cultured vascular endothelial cells [33]. It is suggested that the mechanisms underlying transcriptional induction of *MT-1A* and *MT-2A* by As35 and Sb35 are similar, but the induction by As35 can occur at lower concentrations because of the higher intracellular accumulation. Although it is certain that the intramolecular elements can modify the bioactivities of organometallic compounds, the mechanisms underlying the modifications remain to be determined.

In the present study, we demonstrate the intracellular signaling pathways for transcriptional induction of endothelial MT isoforms using the organic-inorganic hybrid molecules Sb35 and P35. We found that there are two signaling pathways—the MTF-1–MRE and Nrf2–ARE pathways—that regulate the transcription of endothelial MT. The MTF-1–MRE pathway is essential for induction of all isoforms and the Nrf2–ARE pathway reinforces this induction. In addition, there is a mechanism by which only MT-2A is induced in vascular endothelial cells. It is suggested that MT-1A/E and MT-2A could have different functions. Additionally, proteasome inhibition could be involved in transcriptional regulation by modulating Nrf2 recruitment to AREs in the promoter regions of *MT* genes via Nrf2 acetylation. In addition, MTF-1 is activated by zinc; zinc binds to the zinc finger domain of MTF-1 to recruit transcription factors to the MREs [45,46]. Zinc is the only heavy metal that can induce MTF-1 binding to MREs [47]. Although the origin of zinc, which activates MTF-1 after P35 treatment, is unclear, two mechanisms are possible. First, the zinc ion could be supplied to MTF-1 by intracellular proteins that bind zinc ions nonspecifically. The difference in MTF-1 activating activity between Sb35 and P35 may be due to the different capacity to release zinc from zinc-binding proteins. Second, recently, a family of zinc transporters was identified [48], which are expressed on the transmembrane [49], Golgi apparatus [50], and endoplasmic reticulum [51] and may regulate the intracellular concentration of zinc. It is possible that either Sb35, As35, or P35 activates or induces zinc transporter(s), which releases the zinc ion into the cytoplasm, thereby activating MTF-1. Although the mechanisms for endothelial MT induction remain to be elucidated, our present study revealed the exact role of the Nrf2–ARE pathway in endothelial MT transcriptional induction by using an organometallic compound as an endothelial MT inducer. This suggests that the strategy of bio-organometallics, in which organic-inorganic hybrid molecules are used as tools for analysis of biological functions, is effective to reveal unknown biological mechanisms.

## 4. Experimental Section

### 4.1. Synthesis of Sb35, P35, and As35

Sb35, tris(pentafluorophenyl)arsane (As35), and tris(pentafluorophenyl)phosphane (P35) were synthesized according to previously reported procedures [52,53,54,55].

### 4.2. Cell Culture and Treatment

Bovine aortic endothelial cells (Cell Applications, CA, USA) were cultured at 37 °C in 5% CO_2_ in Dulbecco’s modified Eagle’s medium supplemented with 10% fetal bovine serum (Thermo Fisher Scientific, Waltham, MA, USA) until confluent. The cells were treated with or without Sb35 (10, 50, 100, 150, or 200 µM), As35 (5, 10, 20, or 30 µM), or P35 (5, 10, 20, or 30 µM) at 37 °C for 3, 6, 12, 24, or 48 h in serum-free Dulbecco’s modified Eagle’s medium after washing twice with serum-free Dulbecco’s modified Eagle’s medium. In another experiment, confluent cultures of human brain microvascular endothelial cells were treated with 28 organoantimony compounds (Table 1) at 10 µM for 3 h in HuMedia EG-2 (Kurabo, Osaka, Japan), and *MT-1X* mRNA expression was determined by real-time RT-PCR as described below. The primer pair was 5′-TTGATCGGGAACTCCTGCTTCT-3′ (forward) and 5′-ACACTTGGCACAGCCCACA-3′ (reverse). Since the organometallic compounds used in this study were insoluble in water, they were dissolved in dimethylsulfoxide and then added to the culture medium. The final concentration of dimethylsulfoxide was less than 0.1%.

### 4.3 Transfection

Vascular endothelial cells were cultured and were transfected with small-interfering RNAs (siRNAs; Bioneer, Daejeon, Korea) by using RNAiMAX reagent (Invitrogen, Carlsbad, CA, USA), according to the manufacturer's instructions. After transfection, the cells were treated with Sb35, As35, or P35 for 12 h. Sequences of the sense and antisense strands of siRNAs are as follows: bovine *Nrf2* siRNA, 5′-CCAUUGAUCUCUCUGAUCUdTdT-3′ (sense) and 5′-AGAUCAGAGAGAUCAAUGGGC-3′ (antisense); bovine *MTF-1* siRNA, 5′-GAGAACACUUGCCUUUUCUdTdT-3′ (sense) and 5′-AGAAAAGGCAAGUGUUCUCCG-3′ (antisense). A nonspecific sequence was used as negative control siRNA (Qiagen, Valencia, CA, USA).

### 4.4. Luciferase Assay

MRE and ARE-driven reporter assays by using firefly reporter plasmids pGL4.12-MRE_d4_ and pGL4.12-ARE_4_ × 3 [56,57] were performed as described previously [58]. After transfection with the MRE or ARE reporter vector, vascular endothelial cells were treated with Sb35, P35, or CdCl_2_ for 12 h. The cells were then lysed, and luciferase activity was measured using Dual-Luciferase Reporter Assay System (Promega, Madison, WI, USA) and GloMax 20/20n luminometer (Promega, Madison, WI, USA). Luminescence of pRL-SV40 and pRL-TK (Promega) was used to normalize MRE- and ARE-driven transcriptional activities, respectively.

### 4.5. Real-Time RT-PCR

mRNA expression of endothelial *MT-1A*, *MT-1E*, and *MT-2A* was analyzed by performing real-time RT-PCR. Total RNA was extracted from Sb35-, As35-, or P35-treated vascular endothelial cells that were transfected with or without the siRNAs by using RNeasy Lipid Tissue Mini kit (Qiagen, Valencia, CA, USA). Complementary DNA (cDNA) was synthesized using High-Capacity cDNA Reverse Transcription kit (Applied Biosystems, Foster City, CA, USA). Real-time PCR was performed using Gene Ace SYBR qPCR Mixα (Nippon Gene, Tokyo, Japan), 10 ng cDNA, and 100 nM primers in a StepOnePlus RT-PCR system (Applied Biosystems). Sequences of primer pairs used have been described previously [58].

### 4.6. Western Blot Analysis

Protein expression of Nrf2, glutamate-cysteine ligase, modifier subunit (GCLM), heme oxygenase-1 (HO-1), ubiquitin, and β-actin was analyzed by performing Western blotting, as described previously [58]. The following primary antibodies were used: anti-Nrf2 antibody (H-300; Santa Cruz Biotechnology, Santa Cruz, CA, USA), anti-GCLM antibody (FL-274; Santa Cruz Biotechnology), anti-HO-1 antibody (ADI-SPA-895; Enzo Life Sciences, Farmingdale, NY, USA), anti-ubiquitin monoclonal antibody (ADI-SPA-203; Enzo Life Sciences), and anti-β-actin antibody (A5060; Sigma-Aldrich Chemical, St. Louis, MO, USA). Expression of MT protein was analyzed using a method described by Stitt et al. [59] with a mouse monoclonal anti-MT-1/2 antibody (E9; DAKO, Glostrup, Denmark). An apoprotein of MT was detected in this experiment because metals bound to MT were chelated by EDTA and because thiols of cysteine residues in MT were alkylated by iodoacetamide before performing sodium dodecyl sulfate polyacrylamide gel electrophoresis.

### 4.7. Statistical Analysis

Data were analyzed using one-way analysis of variance followed by Bonferroni-type multiple *t*-test. *p* values less than 0.05 were considered significant.

## Figures and Tables

**Table 1:** Organoantimony compounds used in this study.

No.	Molecular Formula	
Sb13	C_23_H_20_NSb	*N*-Methyl-*Sb*-phenylethynyl-5,6,7,12-tetrahydrodibenz[*c*,*f*][1,5]azastibocine
Sb14	C_24_H_22_NSb	*N*-Ethyl-*Sb*-phenylethynyl-5,6,7,12-tetrahydrodibenz[*c*,*f*][1,5]azastibocine
Sb15	C_25_H_24_NSb	*N*-*iso*-propyl-*Sb*-Phenylethynyl-5,6,7,12-tetrahydrodibenz[*c*,*f*][1,5]azastibocine
Sb16	C_26_H_26_NSb	*N*-2-Methylpropyl-*Sb*-phenylethynyl-5,6,7,12-tetrahydrodibenz[*c*,*f*][1,5]azastibocine
Sb17	C_28_H_28_NSb	*N*-Cyclohexyl-*Sb*-phenylethynyl-5,6,7,12-tetrahydrodibenz[*c*,*f*][1,5]azastibocine
Sb18	C_28_H_22_NSb	*N*-Phenyl-*Sb*-phenylethynyl-5,6,7,12-tetrahydrodibenz[*c*,*f*][1,5]azastibocine
Sb19	C_21_H_20_NSb	*N*-Methyl-*Sb*-phenyl-5,6,7,12-tetrahydrodibenz[*c*,*f*][1,5]azastibocine
Sb20	C_24_H_26_NSb	*N*-*t*-Butyl-*Sb*-phenyl-5,6,7,12-tetrahydrodibenz[*c*,*f*][1,5]azastibocine
Sb22	C_16_H_18_NSb	*N*-Methyl-*Sb*-methyl-5,6,7,12-tetrahydrodibenz[*c*,*f*][1,5]azastibocine
Sb23	C_19_H_26_NSb	*N*-Methyl-*Sb*-trimethylsilylmethyl-5,6,7,12-tetrahydrodibenz[*c*,*f*][1,5]azastibocine
Sb24	C_19_H_24_NSb	*N*-*t*-Butyl-*Sb*-methyl-5,6,7,12-tetrahydrodibenz[*c*,*f*][1,5]azastibocine
Sb26	C_21_H_21_O_3_Sb	Tris(4-methoxylphenyl)stibane
Sb29	C_21_H_21_Sb	Tris(4-methylphenyl)stibane
Sb30	C_21_H_21_Sb	Tris(3-methylphenyl)stibane
Sb31	C_21_H_21_Sb	Tris(2-methylphenyl)stibane
Sb32	C_27_H_33_Sb	Tris(2,4,6-trimethylphenyl)stibane
Sb33	C_18_H_12_F_3_Sb	Tris(4-fluorophenyl)stibane
Sb34	C_18_H_12_Cl_3_Sb	Tris(4-chlorophenyl)stibane
Sb35	C_18_F_15_Sb	Tris(pentafluorophenyl)stibane
Sb37	C_24_H_15_S_3_Sb	Tris(1-benzothiophen-2-yl)stibane
Sb38	C_24_H_15_O_3_Sb	Tris(2-benzofuranyl)stibane
Sb40	C_21_H_12_F_9_Sb	Tris[(4-trifluoromethyl)phenyl]stibane
Sb41	C_27_H_27_O_6_Sb	Tris(4-ethoxycarbonylphenyl)stibane
Sb42	C_27_H_36_N_3_Sb	Tris[2-(*N*,*N*-dimethylaminomethyl)phenyl]stibane
Sb43	C_24_H_27_O_3_Sb	Tris[2-(methoxymethyl)phenyl]stibane
Sb44	C_24_H_27_S_3_Sb	Tris[2-(methylsulfanylmethyl)phenyl]stibane
Sb46	C_18_H_15_Cl_2_Sb	Triphenylantimony dichloride
Sb48	C_22_H_21_O_4_Sb	Triphenylantimony diacetate

## Supplementary Materials

Supplementary materials can be found at www.mdpi.com/1422-0067/17/9/1381/s1.

## Abbreviations

ARE	Antioxidant Response Element
As35	Tris(pentafluorophenyl)arsane
GAPDH	Glyceraldehydes-3-Phosphate Dehydrogenase
GCLM	Glutamate-Cysteine Ligase, Modifier subunit
HO-1	Heme Oxygenase-1
MRE	Metal Responsive Element
MTF-1	Metal response element-binding Transcription Factor-1
Nrf2	Nuclear Factor-erythroid 2-related factor 2
P35	Tris(pentafluorophenyl)phosphane
Sb35	Tris(pentafluorophenyl)stibane
siRNA	Small interfering RNA
