# Supplementary material for: Transcriptional Induction of Metallothionein by Tris(pentafluorophenyl)stibane in Cultured Bovine Aortic Endothelial Cells
Source: Int J Mol Sci. 2016 Aug 23;17(9):1381. doi: 10.3390/ijms17091381 (PMC5037661; doi:10.3390/ijms17091381)
Supplement: Supplementary file 1 [file ijms-17-01381-s001.pdf]

Supplementary Materials: Transcriptional Induction of Metallothionein by Tris(pentafluorophenyl)stibane in Cultured Bovine Aortic Endothelial Cells

Tomoya Fujie, Masaki Murakami, Eiko Yoshida, Shuji Yasuike, Tomoki Kimura, Yasuyuki Fujiwara, Chika Yamamoto and Toshiyuki Kaji

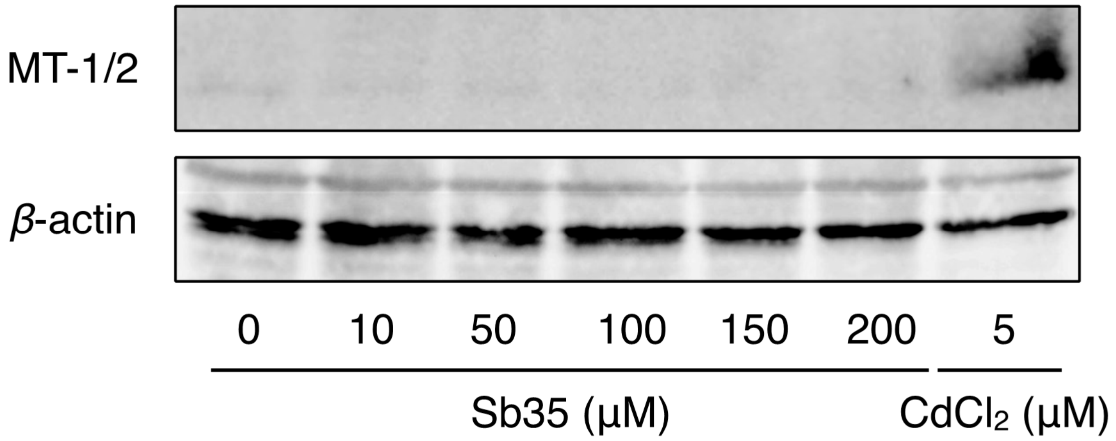

Figure S1. Expression of metallothionein-1/2 protein (MT-1/2). Vascular endothelial cells were treated with or without Sb35 (10, 50, 100, 150, or 200 μM) or $CdCl_2$ (5 μM) for 24 h.

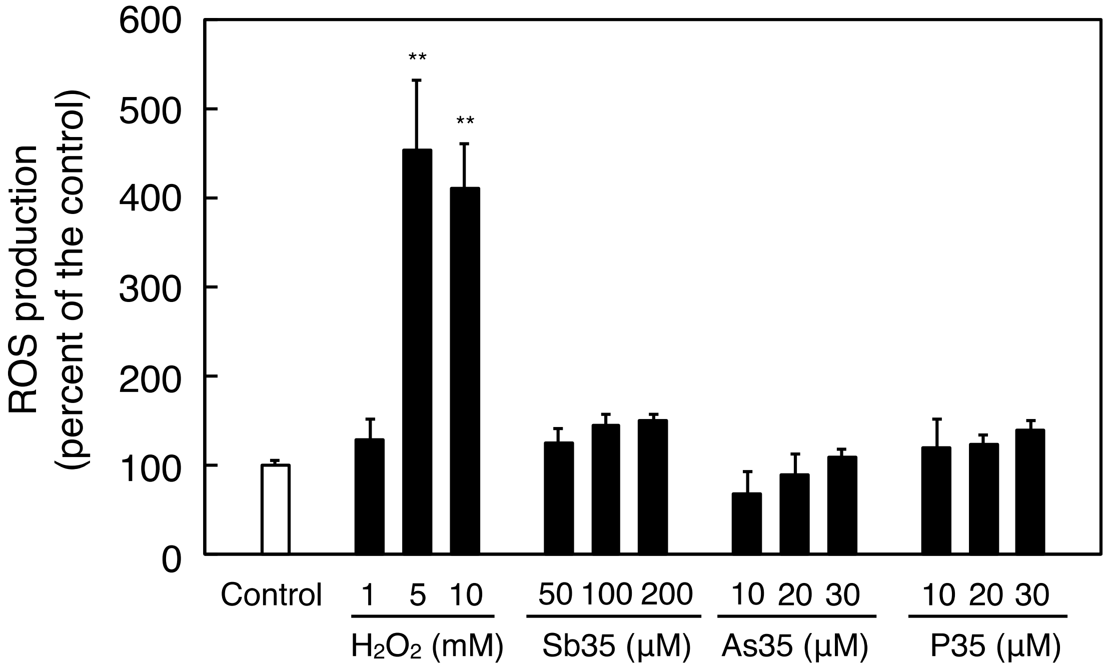

Figure S2. Production of reactive oxygen species (ROS) by Sb35, As35, or P35 in vascular endothelial cells. Vascular endothelial cells were treated with or without Sb35 (50, 100, or 200 μM), As35 (10, 20, or 30 μM), P35 (10, 20, or 30 μM), or hydrogen peroxide (H2O2) (1, 5, or 10 mM) for 3 h after treatment with 10 μM dihydroethidium for 1 h. Data are represented as mean ± SE of six representative samples, with each sample obtained from six independent experiments. ** $p < 0.01$ indicates significantly different from the control.
